# Low expression of SLC34A1 is associated with poor prognosis in clear cell renal cell carcinoma

**DOI:** 10.1186/s12894-023-01212-x

**Published:** 2023-03-28

**Authors:** Jiechuan Qiu, Zicheng Wang, Yingkun Xu, Leizuo Zhao, Peizhi Zhang, Han Gao, Qingliang Wang, Qinghua Xia

**Affiliations:** 1 Department of Urology, Shandong Provincial Hospital Affiliated to Shandong First Medical University, 9677 Jingshidong Road, Jinan City, 250001 Shandong Province China; 2 Department of Breast and Thyroid Surgery, The First Affiliated Hospital of Chongqing Medical University, Chongqing, 400042 China; 3 Department of Urology, Shandong Provincial Hospital, Cheeloo College of Medicine, Shandong University, Jinan, 250021 China; 4 Department of Urology, Dongying People’s Hospital, Dongying, 257000 China

**Keywords:** SLC34A1, Clear cell renal cell carcinoma, Diagnosis, Prognostic marker

## Abstract

**Objective:**

Clear cell renal cell carcinoma (ccRCC) is a malignant renal tumor that is highly prone to metastasis and recurrence. The exact pathogenesis of this cancer is still not well understood. This study aimed to identify novel hub genes in renal clear cell carcinoma and determine their diagnostic and prognostic value.

**Methods:**

Intersection genes were obtained from multiple databases, and protein–protein interaction analysis and functional enrichment analysis were performed to identify key pathways related to the intersection genes. Hub genes were identified using the cytoHubba plugin in Cytoscape. GEPIA and UALCAN were utilized to observe differences in mRNA and protein expression of hub genes between KIRC and adjacent normal tissues. The Wilcoxon rank sum test was used to analyze hub gene levels between paired KIRC and matched non-cancer samples. IHC results were obtained from the HPA online database, and according to the median gene expression level, they were divided into a high-expression group and a low-expression group. The correlation of these groups with the prognosis of KIRC patients was analyzed. Logistic regression and the Wilcoxon rank sum test were used to test the relationship between SLC34A1 level and clinicopathological features. The diagnostic value of SLC34A1 was evaluated by drawing the receiver operating characteristic (ROC) curve and calculating the area under the curve (AUC). Cox regression analysis was used to analyze the relationship between clinicopathological features, SLC34A1 expression, and KIRC survival rate. LinkedOmics was used to obtain the genes most related to SLC34A1 and their functional enrichment. Genetic mutations and methylation levels of SLC34A1 in KIRC were obtained from the cBioPortal website and the MethSurv website, respectively.

**Results:**

Fifty-eight ccRCC differential genes were identified from six datasets, and they were mainly enriched in 10 functional items and 4 pathways. A total of 5 hub genes were identified. According to the GEPIA database analysis, low expression of SLC34A1, CASR, and ALDOB in tumors led to poor prognosis. Low expression of SLC34A1 mRNA was found to be related to clinicopathological features of patients. SLC34A1 expression in normal tissues could accurately identify tumors (AUC 0.776). SLC34A1 was also found to be an independent predictor of ccRCC in univariate and multivariate Cox analyses. The mutation rate of the SLC34A1 gene was 13%. Eight of the 10 DNA methylated CpG sites were associated with the prognosis of ccRCC. SLC34A1 expression in ccRCC was positively correlated with B cells, eosinophils, neutrophils, T cells, TFH, and Th17 cells, and negatively correlated with Tem, Tgd, and Th2 cells.

**Conclusion:**

The expression level of SLC34A1 in KIRC samples was found to be decreased, which predicted a decreased survival rate of KIRC. SLC34A1 may serve as a molecular prognostic marker and therapeutic target for KIRC patients.

**Supplementary Information:**

The online version contains supplementary material available at 10.1186/s12894-023-01212-x.

## Introduction

Renal cell carcinoma is one of the most prevalent malignancies, accounting for 5% and 3% of all malignant tumors in men and women, respectively [1]. Clear cell renal cell carcinoma (ccRCC) is the most common subtype, comprising approximately 80% of all cases [2]. Despite the application of immunotherapy and targeted therapies, the survival rate of patients with advanced and metastatic ccRCC remains poor due to the development of drug resistance [3]. Therefore, identifying updated prognostic markers is crucial for developing new drugs for advanced-stage and metastatic ccRCC.

In recent years, bioinformatics analysis based on gene expression microarray has been useful in identifying key genes and central networks involved in tumorigenesis and development. Screening biological indicators of cancer treatment and prognosis is a promising approach. Researchers are increasingly using the GEO database to identify key genes related to cancer and make breakthroughs in cancer treatment. For example, Liu et al. [4] identified five key genes related to thyroid cancer after thorough analysis of the GEO database. Similarly, Zhou et al. [5] identified 15 hub genes strongly enriched in multiple pathways related to hepatocellular carcinoma through bioinformatics analysis. Some scholars have also applied bioinformatics to diagnose and predict prognosis of renal cell carcinoma, finding potential targets for ccRCC diagnosis, therapy, and prognosis [6].

Building on these studies, we identified hub genes of ccRCC by analyzing gene expression profiles from the GEO database, selecting SLC34A1 for further investigation. In this study, we explore the relationship between SLC34A1 and immune cell infiltration and its diagnostic and prognostic value in ccRCC. We also investigate the relationship between SLC34A1-related genes and the prognosis of ccRCC, as well as gene mutation and DNA methylation, providing new target genes for future transformation and clinical treatment. The study workflow is illustrated in Additional file 1: Fig. S1.

## Materials and methods

### Downloading raw data

We downloaded six gene expression profile datasets (including GSE66272, GSE53757, GSE68417, GSE168845, GSE96574, and GSE40435) from the GEO database (https://www.ncbi.nlm.nih.gov/geo/). The GSE66272 dataset contains 26 ccRCC samples and 26 normal tissue samples (a pair of sarcoma samples, GSM1618417 and GSM1618418, were excluded). The GSE53757 dataset consists of 72 ccRCC samples and 72 adjacent tissue samples. The GSE68417 dataset includes 14 ccRCC samples and 29 adjacent tissue samples. The GSE168845 dataset contains 4 ccRCC samples and 4 normal tissue samples. The GSE96574 dataset contains 5 ccRCC samples and 5 adjacent normal tissue samples. The GSE40435 dataset includes 101 ccRCC samples and 101 adjacent tissue samples. We obtained the original TCGA-KIRC file from the TCGA database (https://portal.gdc.cancer.gov/).

### Differential gene detection

We processed the downloaded raw data using the annotation package in the R software (version 3.6.3; https://www.r-project.org/). The gene probe names in the original data files were changed to the international standard name using the Perl language. The R software package for gene differential expression analysis is available from the Bioconductor website (http://www.bioconductor.org/). We used the limma package to preprocess the gene expression data and obtain the differentially expressed genes (DEGs) in the gene expression data file [7]. We set *P* < 0.05 and |log2 fold change|≥ 2 as the screening threshold for DEGs. We obtained the intersection sets of up-regulated and down-regulated DEGs in ccRCC from the six datasets using the TBtools tool [8]. Volcanic maps represent the DEGs of six data sets.

### Functional analysis and PPI network construction

We used online biological tools to investigate the biological process (BP), molecular function (MF), and cellular component (CC) of the DEGs. We used DAVID 6.8 (https://david.ncifcrf.gov/) for GO enrichment analysis and KEGG pathway analysis [9, 10]. KEGG pathways were derived from the KEGG database, which was initiated in 1995 under the Japanese Human Genome Project [11]. Its main role is to systematically analyze gene function and link genomic information with higher-order functional information [12]. The KEGG database integrates various biological objects classified as systematic, genomic, chemical, and health information [13]. Each object (database entry) is identified by a KEGG identifier (kid). We set *P* < 0.05 and FDR < 0.05 as critical thresholds for GO and KEGG pathway analysis. We used the ggplot2 package of R software to create a histogram of the central path. We constructed a PPI network of the DEGs using the online website STRING (http://string-db.org) and the Cytoscape program (version 3.9.0, http://www.cytoscape.org/). We employed the cytoHubba plug-in in Cytoscape software to screen central genes.

### Verification of protein expression

We analyzed and verified the protein expression level of hub genes using the UALCAN online tool (http://ualcan.path.uab.edu/index.html) and the HPA database (http://www.proteinatlas.org/) [14, 15].

### Expression and survival analysis of hub genes

We used the GEPIA web platform (http://gepia.cancer-pku.cn/) based on TCGA and GTEx datasets to analyze the expression of hub genes in tumor and normal tissues and the relationship between hub gene expression and overall survival (OS) and disease-free survival (DFS). We verified the analysis results obtained by the GEPIA online tool using the UALCAN online tool and the ggplot2 package in R software.

### Clinical relevance, diagnostic ROC, and univariate/multivariate cox risk regression analysis

The relationship between SLC34A1 and clinical variables, such as age, gender, pathological stage, histological grade, T stage, N stage, and M stage, was investigated using the R software package, ggplot2. The diagnostic value of SLC34A1 in ccRCC was analyzed using the ROC curve, which was created using the pROC software package in R [16]. To identify the independent prognostic factors of ccRCC, univariate/multivariate Cox risk regression analysis was conducted on SLC34A1 and seven clinical factors (age, grade, pathological stage, histological grade, gender, T stage, M stage, and N stage) using the R software survival package [17]. The clinical data were obtained from the TCGA database.

### Genes associated with SLC34A1

The SLC34A1 coexpression network in ccRCC was evaluated using the LinkFinder module of the LinkedOmics database (http://www.linkedomics.org/login.php) [18]. The volcano map depicted genes that were related to SLC34A1 expression, and the heat map displayed the top 50 genes that were positively and negatively correlated with SLC34A1. GO term enrichment and KEGG pathway analyses of SLC34A1-related genes were conducted to study their functions. The GEPIA online tool was used to verify the correlation and analyze the overall survival of the five co-expressed genes most related to SLC34A1.

### Genetic changes in patients with ccRCC

The genome map of SLC34A1 in ccRCC was analyzed using two data sets, TCGA Firehose Legacy and UTokyo Net Genet 2013, in the cBioPortal database (http://www.cbioportal.org/) [19, 20]. The association between changes in SLC34A1 and major carcinogenic drivers was assessed.

### The relationship between the methylation level of the SLC34A1 gene and prognosis and the relationship between SLC34A1 and immune cells in patients with ccRCC

The DNA methylation sites of SLC34A1 in the TCGA database were obtained using the Methsurv database (https://biit.cs.ut.ee/methsurv/) [21]. The prognostic value of CpG methylation in SLC34A1 was evaluated using overall survival as the survival outcome. The expression of SLC34A1 phosphoprotein was analyzed using the CPTAC online tool [22]. The infiltration relationship between SLC34A1 and immune cells was analyzed using the ssGSEA algorithm in the GSVA package. The statistical method used was Spearman, and the threshold was set at *P*< 0.05.

### Real-time RT-PCR assay

From 2019 to 2021, twenty pairs of KIRC tissues and matched normal tissues were collected from patients who underwent surgery at Shandong Provincial Hospital. All patients were fully informed of the study's purpose, and written consent was obtained. The ethical requirements of the Shandong Provincial Hospital Ethics Committee and the Helsinki Declaration were strictly followed. Total RNA was extracted from tissue lysates using the AG RNAex Pro Reagent (Accurate Biotechnology) and reverse transcribed using the Evo M-MLV RT Premix (Accurate Biotechnology). The SYBR^®^ Green Premix Pro Taq HS qPCR (Accurate Biotechnology) kit was used to perform qRT-PCR assay, and the LightCycle 480 II (Roche) was used to amplify the samples. The following primers were used: GAPDH-F: GGAGCGAGATCCCTCCAAAAT, GAPDH-R: GGCTGTTGTCATACTTCTCATGG, SLC34A1-F: GTTGTCCTACGGAGAGAGGC, SLC34A1-R: GGAAGGCATAGGCAGAGGTC.

### Immunohistochemical staining

Tumor tissue and matching normal kidney tissue were collected from 4 patients with ccRCC who underwent surgery at Shandong Provincial Hospital between February 2020 and June 2021. Each patient provided informed consent. The sections were dewaxed in xylene, rehydrated in graded ethanol, and underwent antigen retrieval in sodium citrate buffer (pH 6.0) at 95 °C for 20 min. After quenching endogenous peroxidase activity with 3% H2O2 and blocking non-specific binding with 1% bovine serum albumin buffer, the sections were incubated overnight with the designated primary antibody at 4 °C. Following repeated washing, the sections were treated with HRP-conjugated secondary antibody at room temperature for 40 min and stained with DAB.

### Statistical method

The Wilcox rank sum test was used to compare multiple groups of variables. The Spearman correlation coefficient was applied to evaluate the genes related to SLC34A1 expression. The Spearman method was used to analyze the correlation coefficient of immune infiltration. *P* < 0.05 was set as the statistically significant level.

## Results

### Identification of differentially expressed genes (DEGs)

**Fig. 1:**
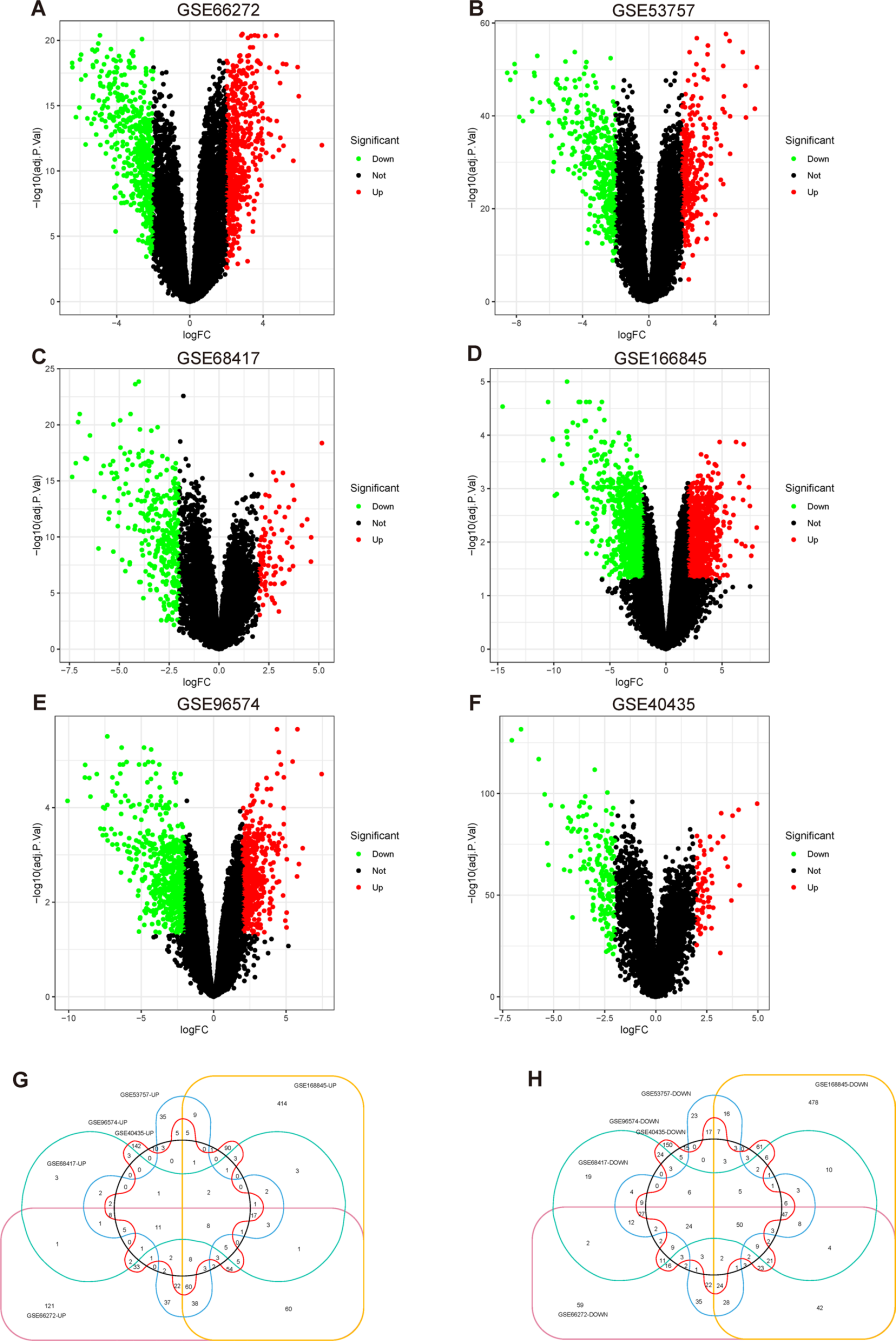
Volcano and Venn Maps of DEGs. Volcano maps were generated for each dataset, including GSE66272 (**A**), GSE53757 (**B**), GSE68417 (**C**), GSE168845 (**D**), GSE96574 (**E**), and GSE40435 (**F**). The x-axis represents logFC and the y-axis represents − log10(*P* value). Venn diagrams were generated to identify common up-regulated (**G**) and down-regulated (**H**) DEGs across the two datasets

The limma package was used to process the datasets, and DEGs were obtained in each of the six datasets based on the screening criteria (*P* < 0.05 and |log2 FC|≥ 2). The resulting DEGs are presented in Table 1. In the GSE66272 dataset, we identified a total of 1014 DEGs, comprising 515 upregulated and 499 downregulated DEGs (Fig. 1A). In the GSE53757 dataset, we identified 686 DEGs, comprising 294 upregulated and 392 downregulated DEGs (Fig. 1B). In the GSE68417 dataset, we identified 424 DEGs, comprising 92 upregulated and 332 downregulated DEGs (Fig. 1C). In the GSE168845 dataset, we identified 1674 DEGs, comprising 800 upregulated and 874 downregulated DEGs (Fig. 1D). In the GSE96574 dataset, we identified 1096 DEGs, comprising 496 upregulated and 600 downregulated DEGs (Fig. 1E). In the GSE40435 dataset, we identified 236 DEGs, comprising 71 upregulated and 165 downregulated DEGs (Fig. 1F). The DEGs of the two groups of sample data in the six datasets are displayed on volcano plots. We intersected the upregulated genes in all six datasets and obtained eight upregulated DEGs (Fig. 1G). Similarly, we intersected the downregulated genes in all six datasets and obtained 50 downregulated DEGs (Fig. 1H).

**Table 1:** The number of DEGs in GEO data sets

GEO	Normal	Tumor	Number of DEGs	Number of up-regulated genes	Number of genes down-regulated
GSE66272	26	26	1014	515	499
GSE53757	72	72	686	294	392
GSE68417	29	14	424	92	332
GSE168845	4	4	1674	800	874
GSE96574	5	5	1096	496	600
GSE40435	101	101	236	71	165

### Functional and PPI analysis of DEGs and identification of core genes

**Fig. 2:**
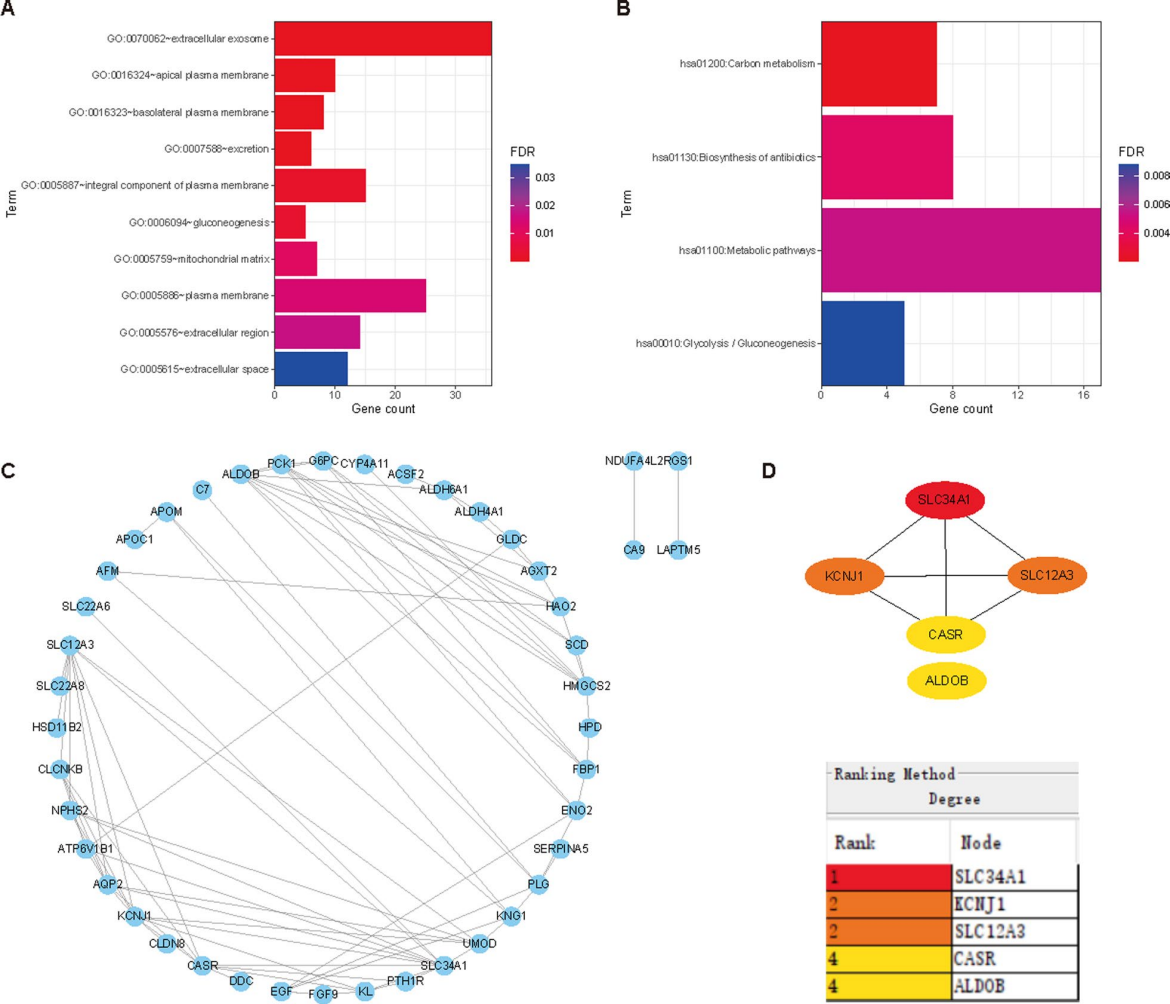
Functional and PPI network analysis of DEGs. **A** GO enrichment analysis of DEGs; **B** KEGG pathway analysis of DEGs. **C** PPI network of all DEGs built using Cytoscape software. **D** Identification of the top five central DEGs using the cytoHubba plugin, with grades indicated by varying degrees of color (from red to yellow)

To investigate the functions of the 58 DEGs identified, we performed GO and KEGG analyses using the DAVID online service (threshold: *P* < 0.05, FDR < 0.05, and count ≥ 5). We sorted the results of functional enrichment and pathway analysis and presented them in Tables 2 and 3, respectively. We used the ggplot2 package in R software to visualize the analysis results. The GO enrichment analysis comprised two main functions: biological processes and cellular components. The cellular components included "extracellular exosome," "apical plasma membrane," "basolateral plasma membrane," "integral component of plasma membrane," "mitochondrial matrix," "plasma membrane," "extracellular region," and "extracellular space." The biological processes mainly included "excretion" and "gluconeogenesis" (Fig. 2A). According to KEGG pathway analysis, DEGs were significantly enriched in Carbon metabolism, Antibiotic biosynthesis, Metabolic pathways, and Glycolysis/Gluconeogenesis (Fig. 2B). We obtained the protein–protein interaction network of DEGs using the STRING online platform and displayed the results using Cytoscape software (Fig. 2C). We then identified the top five hub genes in all DEGs using the Degree algorithm of the cytoHubba plug-in in Cytoscape software: SLC34A1, KCNJ11, SLC12A3, CASR, and ALDOB (Fig. 2D). All these central genes were downregulated in ccRCC.

**Table 2:** GO analysis of intersecting genes

Category	Term	Count	*P* value	FDR
GOTERM_CC_DIRECT	GO:0070062 ~ extracellular exosome	36	3.28E−15	3.38E−13
GOTERM_BP_DIRECT	GO:0007588 ~ excretion	6	1.26E−07	5.91E−05
GOTERM_CC_DIRECT	GO:0016324 ~ apical plasma membrane	10	2.74E−07	1.41E−05
GOTERM_CC_DIRECT	GO:0016323 ~ basolateral plasma membrane	8	1.42E−06	4.88E−05
GOTERM_BP_DIRECT	GO:0006094 ~ gluconeogenesis	5	1.27E−05	0.002974682
GOTERM_CC_DIRECT	GO:0005887 ~ integral component of plasma membrane	15	8.72E−05	0.002246264
GOTERM_CC_DIRECT	GO:0005759 ~ mitochondrial matrix	7	5.36E−04	0.011034772
GOTERM_CC_DIRECT	GO:0005886 ~ plasma membrane	25	7.95E−04	0.013652422
GOTERM_CC_DIRECT	GO:0005576 ~ extracellular region	14	0.001159845	0.017066292
GOTERM_CC_DIRECT	GO:0005615 ~ extracellular space	12	0.0027051 95	0.034829387

**Table 3:** KEGG pathway analysis of intersection genes

Term	Count	*P* value	FDR
hsa01200:Carbon metabolism	7	2.24E−05	0.002062412
hsa01130:Biosynthesis of antibiotics	8	9.32E−05	0.004287886
hsa01100:Metabolic pathways	17	1.77E−04	0.005434928
hsa00010:Glycolysis/Gluconeogenesis	5	3.83E−04	0.008808888

### mRNA expression levels of hub genes

**Fig. 3:**
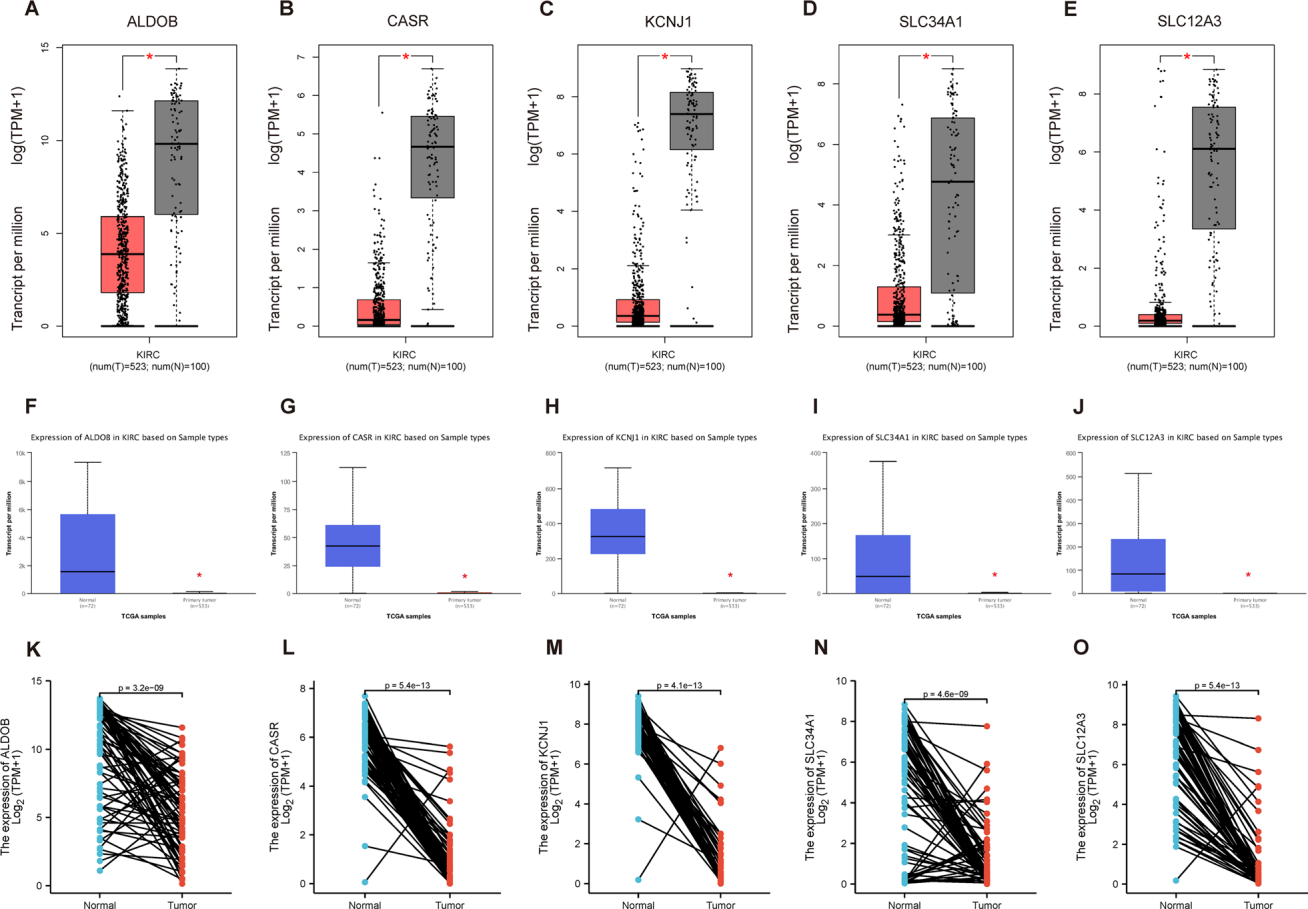
mRNA expression levels of hub genes in ccRCC. **A**–**E** show results obtained from GEPIA for **A** ALDOB, **B** CASR, **C** KCNJ1, **D** SLC34A1, and **E** SLC12A3. **F**–**J** show results obtained from UALCAN for the same genes. **K**–**O** show results obtained from the TCGA dataset. Statistically significant differences (*P < 0.001) are indicated

The authors analyzed the mRNA expression levels of the five hub genes using the GEPIA online analysis tool. The analysis showed a significant decrease in the expression of all five hub genes in ccRCC (Fig. 3A–E). To increase the reliability of the results, the expression of the hub genes was verified using the UALCAN online tool and the TCGA data set, which confirmed the significant downregulation of these genes in ccRCC (Fig. 3F–O). These results provide further evidence supporting the role of the hub genes in ccRCC pathogenesis.

### Protein expression of hub genes

**Fig. 4:**
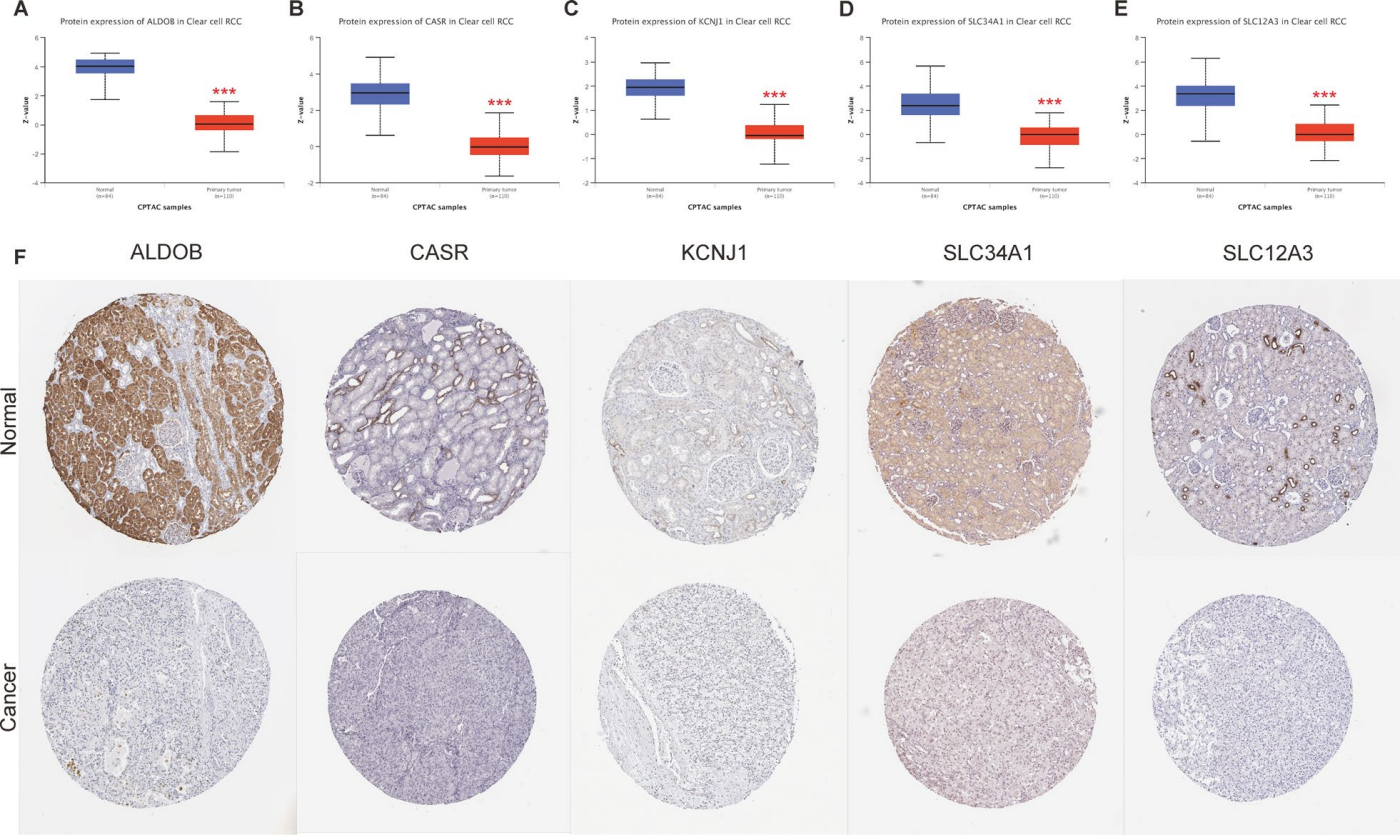
Protein expression levels of hub genes in ccRCC. **A**–**E** show the results obtained through UALCAN. **A** ALDOB, **B** CASR, **C** KCNJ1, **D** SLC34A1, **E** SLC12A3. ***P < 0.001 indicates statistical significance. **F** Representative tissue microarray (TMA) slides showing protein staining of hub genes obtained by HPA

To investigate whether the protein expression levels of hub genes were also altered in tumor tissues, we used the UALCAN tool to analyze the protein expression of these genes. The results showed that the protein expression levels of hub genes were significantly reduced in ccRCC when compared to normal tissues (Fig. 4A–E). We further compared the protein expression levels in cancer and normal tissues using the Human Protein Atlas (HPA). Staining intensity and quantity were evaluated as indicators of protein expression levels in tumor and normal tissues. Our analysis revealed that compared to normal tissues, the protein expression levels of ALDOB, CASR, KCNJ1, SLC34A1, and SLC12A3 were significantly lower in ccRCC tissues, as evidenced by reduced staining, intensity, and quantity (Fig. 4F).

### Prognostic analysis of hub genes in ccRCC

**Fig. 5:**
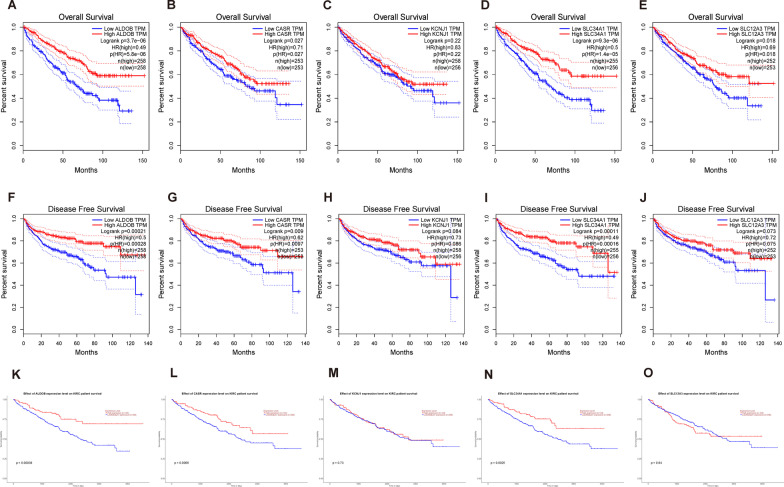
Association between central gene expression levels and overall survival (OS) and disease-free survival (DFS). **A**–**E** show OS results obtained through the GEPIA online platform for ALDOB, CASR, KCNJ1, SLC34A1, and SLC12A3, respectively. **F**–**J** show DFS results obtained through GEPIA for the same genes. **K**–**O** show results obtained through the UALCAN online platform for the same genes. Statistically significant results are indicated by *P* < 0.05

The prognostic value of the five hub genes was evaluated using the GEPIA online tool. Among them, ALDOB, CASR, and SLC34A1 were found to have the potential to predict the prognosis of patients with ccRCC. The results showed that low expression of ALDOB, CASR, and SLC34A1 in patients with ccRCC was associated with lower overall survival (Fig. 5A, B, D), while no significant difference was observed in the other hub genes (Fig. 5C–E). Similarly, individuals with poor expression of the three hub genes also had lower disease-free survival rates (Fig. 5F, G, I), while no significant difference was observed among the other hub genes (Fig. 5H–J). The results of overall survival for the three hub genes were consistent with those obtained using UALCAN (Fig. 5K–O).

### Clinical features of ccRCC

We obtained clinical information and gene expression profile matrices for 539 primary tumors and 72 normal ccRCC samples from the TCGA database. The data includes patients' age, gender, grade, stage, distant metastasis, T classification, and lymph node metastasis (Table 4).

**Table 4:** Clinical features of ccRCC from the data set of TCGA database

Characteristic	Levels	Overall
n		539
Age, n (%)	< = 60	269 (49.9%)
	> 60	270 (50.1%)
Gender, n (%)	Female	186 (34.5%)
	Male	353 (65.5%)
Pathologic stage, n (%)	Stage I	272 (50.7%)
	Stage II	59 (11%)
	Stage III	123 (22.9%)
	Stage IV	82 (15.3%)
Histologic grade, n (%)	G1	14 (2.6%)
	G2	235 (44.3%)
	G3	207 (39%)
	G4	75 (14.1%)
T stage, n (%)	T1	278 (51.6%)
	T2	71 (13.2%)
	T3	179 (33.2%)
	T4	11 (2%)
N stage, n (%)	N0	241 (93.8%)
	N1	16 (6.2%)
M stage, n (%)	M0	428 (84.6%)
	M1	78 (15.4%)

### Clinical features, diagnosis, and prognostic value of SLC34A1

**Fig. 6:**
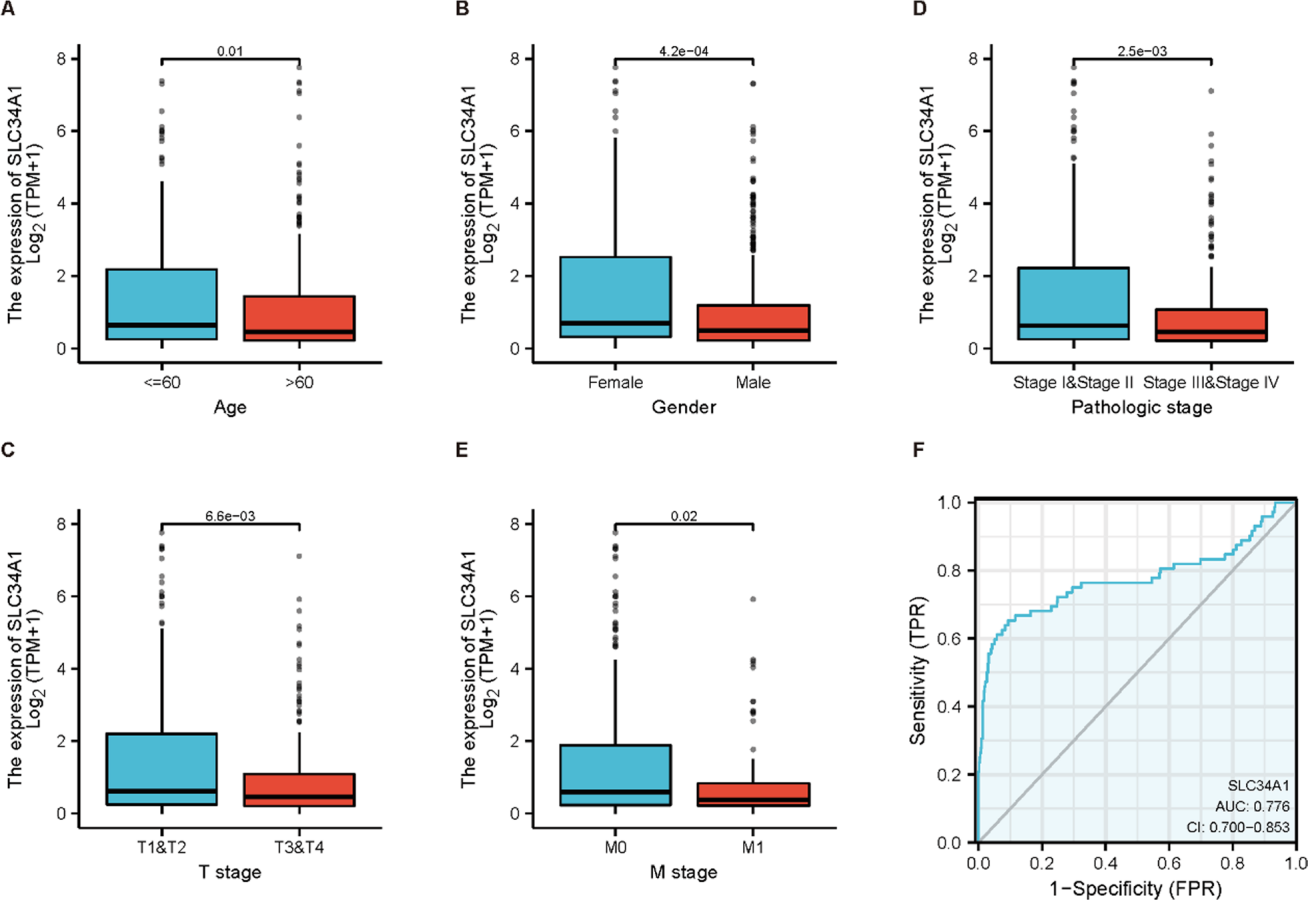
This shows the association between SLC34A1 expression and clinical characteristics, as well as the diagnostic value of SLC34A1. **A**–**E** The expression of SLC34A1 was significantly correlated with age (*P* = 0.01), gender (*P* = 4.2e−04), pathological stage (*P* = 2.5e−03), T stage (*P* = 6.6e−03), and M stage (*P* = 0.02). **F** The diagnostic ROC curve was used to distinguish between tumor and normal tissue, with an area under the curve (AUC) of 0.776

In patients with ccRCC, lower expression levels of SLC34A1 tend to be associated with more advanced cancer stages. The expression of SLC34A1 is correlated with age (Fig. 6A), gender (Fig. 6B), pathological stage (Fig. 6C), T stage (Fig. 6D), and M stage (Fig. 6E). This suggests that the decreased expression of SLC34A1 may be linked to cancer progression. The diagnostic value of SLC34A1 was evaluated by the diagnostic ROC curve, which showed that the area under the curve for SLC34A1 was 0.776 (Fig. 6F). To determine whether SLC34A1 may be a potential prognostic factor, a Cox regression analysis was performed. Univariate Cox risk regression analysis indicated that age, pathological stage, histological grade, T stage, N stage, M stage, and SLC34A1 expression were all significantly associated with OS of ccRCC (all *P* < 0.001) (Table 5). Multivariate Cox risk regression analysis further revealed that histological grade, M stage, and SLC34A1 expression were significantly associated with the overall survival of ccRCC (all *P* < 0.05).

**Table 5:** Cox regression analysis of prognostic factors in patients with clear cell renal cell carcinoma (univariate and multivariate)

Characteristics	Univariate analysis		Multivariate analysis	
	Hazard ratio (95% CI)	*P* value	Hazard ratio (95% CI)	*P* value
Age	1.765 (1.298–2.398)	< 0.001	1.480 (0.949–2.308)	0.084
Gender	0.930 (0.682–1.268)	0.648		
Pathologic stage	3.946 (2.872–5.423)	< 0.001	1.232 (0.485–3.132)	0.660
Histologic grade	2.702 (1.918–3.807)	< 0.001	1.682 (1.025–2.761)	0.040
T stage	3.228 (2.382–4.374)	< 0.001	1.549 (0.680–3.529)	0.298
N stage	3.453 (1.832–6.508)	< 0.001	1.653 (0.824–3.314)	0.157
M stage	4.389 (3.212–5.999)	< 0.001	2.584 (1.520–4.393)	< 0.001
SLC34A1	0.515 (0.377–0.704)	< 0.001	0.612 (0.389–0.963)	0.034

### Genes associated with SLC34A1 expression

**Fig. 7:**
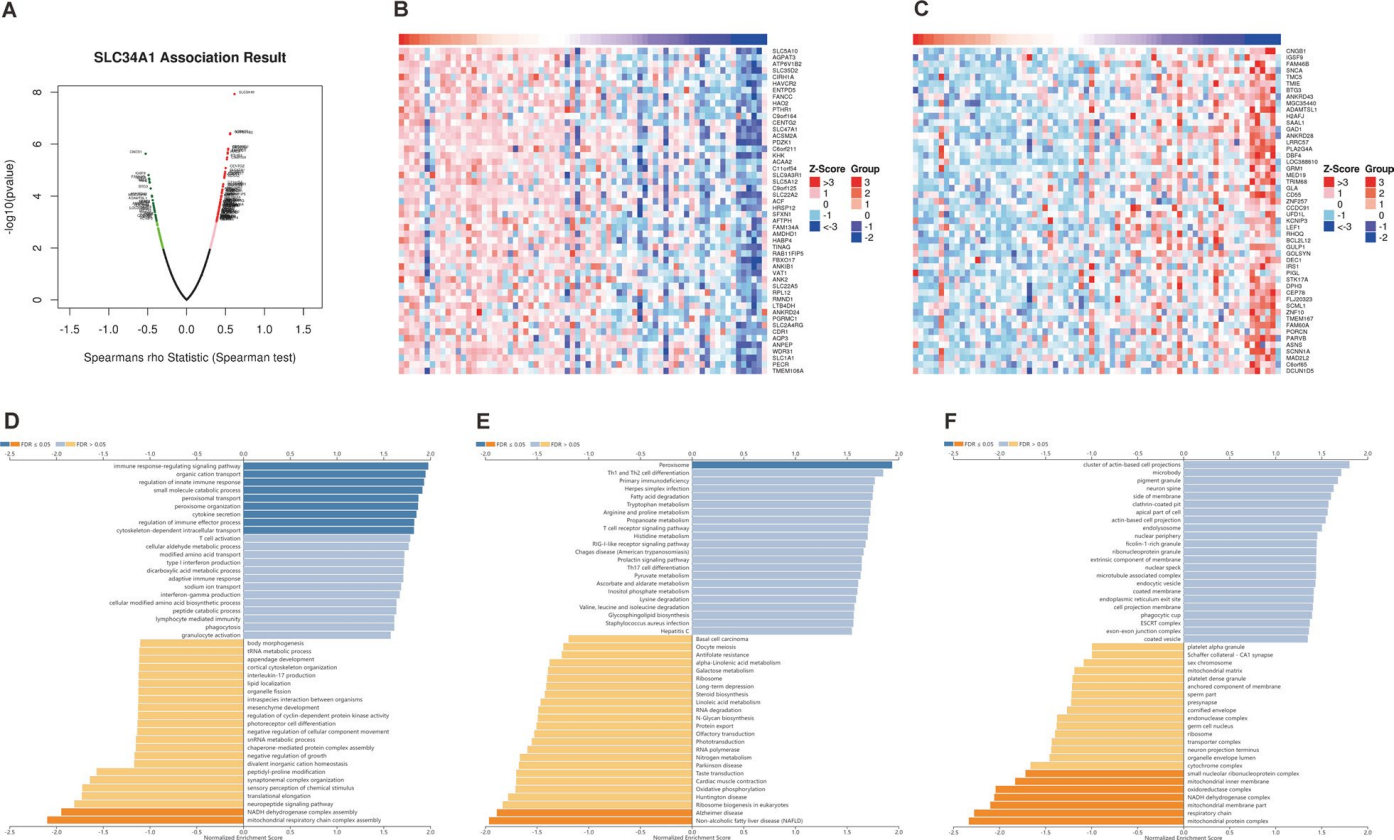
Gene and functional enrichment analysis related to SLC34A1. **A** Volcano plot displaying 3234 genes that were positively correlated with SLC34A1 expression and 2914 genes that were negatively correlated with SLC34A1 expression. **B** Heatmap showing the top 50 genes positively correlated with SLC34A1 expression. **C** Heatmap showing the top 50 genes negatively correlated with SLC34A1 expression. **D** Gene Ontology analysis of SLC34A1-related genes, highlighting biological processes. **E** Gene Ontology analysis of SLC34A1-related genes, highlighting cellular components. **F** Kyoto Encyclopedia of Genes and Genomes (KEGG) pathway analysis of SLC34A1-related genes

Using the LinkedOmics online tool's LinkFinder function module, we identified 3234 genes positively associated with SLC34A1 expression and 2914 genes negatively associated with it in ccRCC (Fig. 7A). The heat map in Fig. 7B shows the top 50 positively associated genes with SLC34A1, while the heat map in Fig. 7C displays the top 50 negatively associated genes. GO enrichment analysis revealed that SLC34A1 and its positive related genes are primarily involved in biological processes such as immune response regulation, organic cation transport, innate immune response regulation, small molecule catabolic process, peroxisomal transport, cytokine secretion, immune effector process regulation, and cytoskeleton-dependent intracellular transport. On the other hand, genes negatively related to SLC34A1 expression are mainly involved in cellular components and biological processes, such as mitochondrial respiratory chain complex assembly, NADH dehydrogenase complex assembly, mitochondrial protein complex, respiratory chain, mitochondrial membrane part, NADH dehydrogenase complex, oxidoreductase complex, mitochondrial inner membrane, and small nuclear ribonucleoprotein complex (Fig. 7D, E). KEGG pathway analysis showed that the positively related genes were mainly concentrated in the peroxisome pathway, while the negatively correlated genes were primarily involved in the Non-alcoholic fatty liver disease (NAFLD) and Alzheimer's disease pathways (Fig. 7F).

**Fig. 8:**
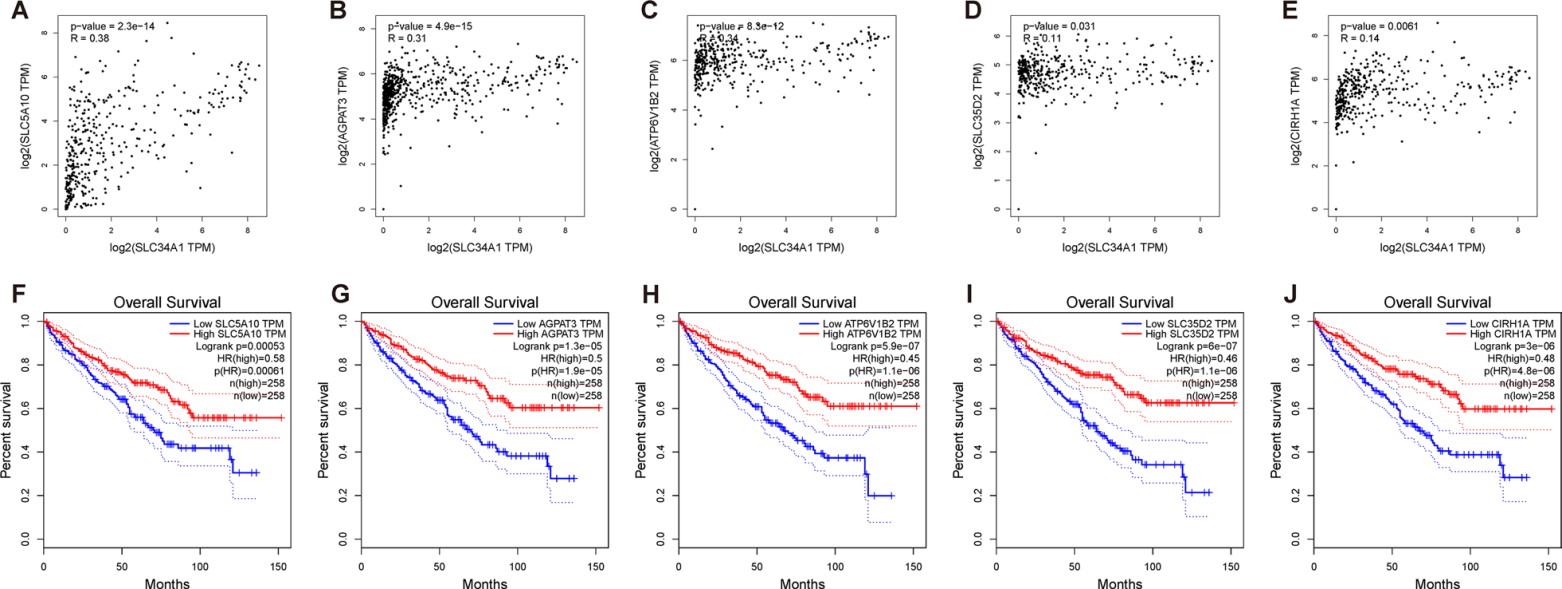
Confirmation of genes with the highest correlation to SLC34A1 expression and their relationship with ccRCC prognosis. **A**–**E** show the correlation of the top five genes: **A** SLC5A10, **B** AGPAT3, **C** ATP6V1B2, **D** SLC35D2, and **E** CIRH1A. **F**–**J** show that SLC5A10, AGPAT3, ATP6V1B2, SLC35D2, and CIRH1A were significantly correlated with better overall survival in patients with ccRCC

The top 5 genes with the strongest correlation to SLC34A1 were selected from the list of genes related to SLC34A1. These five genes were found to be positively correlated with SLC34A1 expression, and they are SLC5A10, AGPAT3, ATP6V1B2, SLC35D2, and CIRH1A. To confirm the correlation between SLC34A1 and these five genes and investigate their impact on the prognosis of ccRCC patients, we used the GEPIA online tool. The analysis revealed that SLC5A10, AGPAT3, ATP6V1B2, SLC35D2, and CIRH1A were significantly correlated with SLC34A1 (Fig. 8A–E), and these five genes were associated with better overall survival in ccRCC patients (Fig. 8F–J).

### SLC34A1 expression changes in patients with ccRCC

**Fig. 9:**
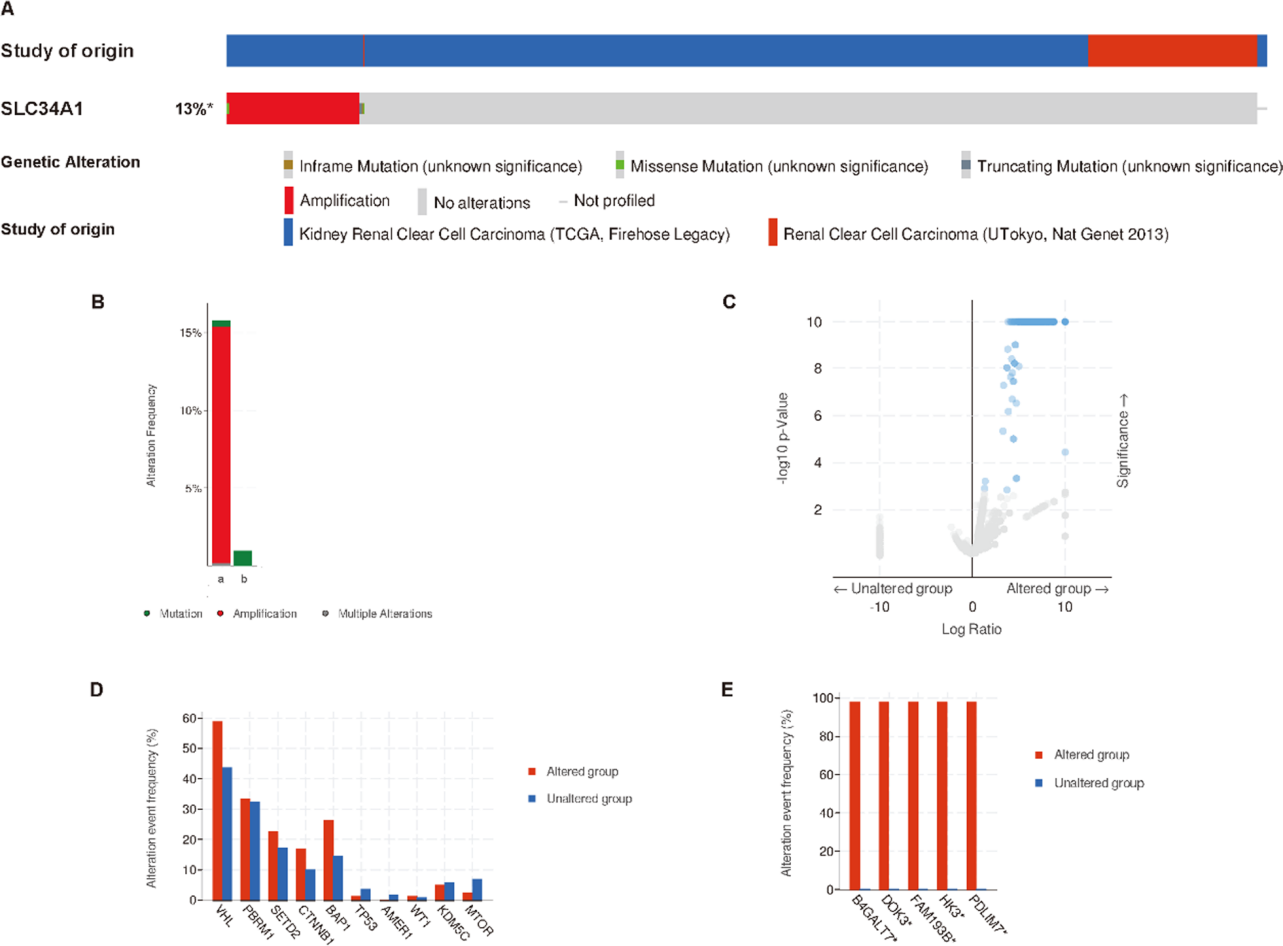
SLC34A1 genetic mutations in ccRCC. **A** The frequency of SLC34A1 mutations among individuals with ccRCC. **B** The incidence of SLC34A1 alterations in ccRCC patients. **C** Gene alteration frequency was significantly higher in the SLC34A1 altered group compared to the SLC34A1 unaltered group. **D** Association between SLC34A1 mutations and changes in major carcinogenic drivers. **E** Top five genes with the highest frequency of alterations in the SLC34A1 change group

We analyzed two ccRCC datasets from the cBioPortal database, UTokyo Nat genet 2013 and TCGA Firehose Legacy, which contained a total of 643 ccRCC patients. The genetic variation frequency of SLC34A1 in ccRCC was found to be 13% (Fig. 9A), with a range of 0.94% (1/106) to 15.82% (84/531) (Fig. 9B). The frequency of gene changes in the SLC34A1 altered group was significantly higher than in the SLC34A1 unchanged group (Fig. 9C). We observed that among the major carcinogenic factors of ccRCC, VHL and BAP1 changed more frequently in ccRCC patients with SLC34A1 than in those without (VHL: 58.82% vs. 43.66%, *P* = 6.343e−3; BAP1: 26.19% vs. 14.31%, *P* = 6.298e−3) (Fig. 9D). The top five genes with the highest frequency in the SLC34A1 alteration group were B4GALT7, DOK3, FAM193B, HK3, and PDLIM7 (Fig. 9E).

### Methylation and phosphoprotein expression of SLC34A1 in ccRCC and its correlation with immune cell infiltration

**Fig. 10:**
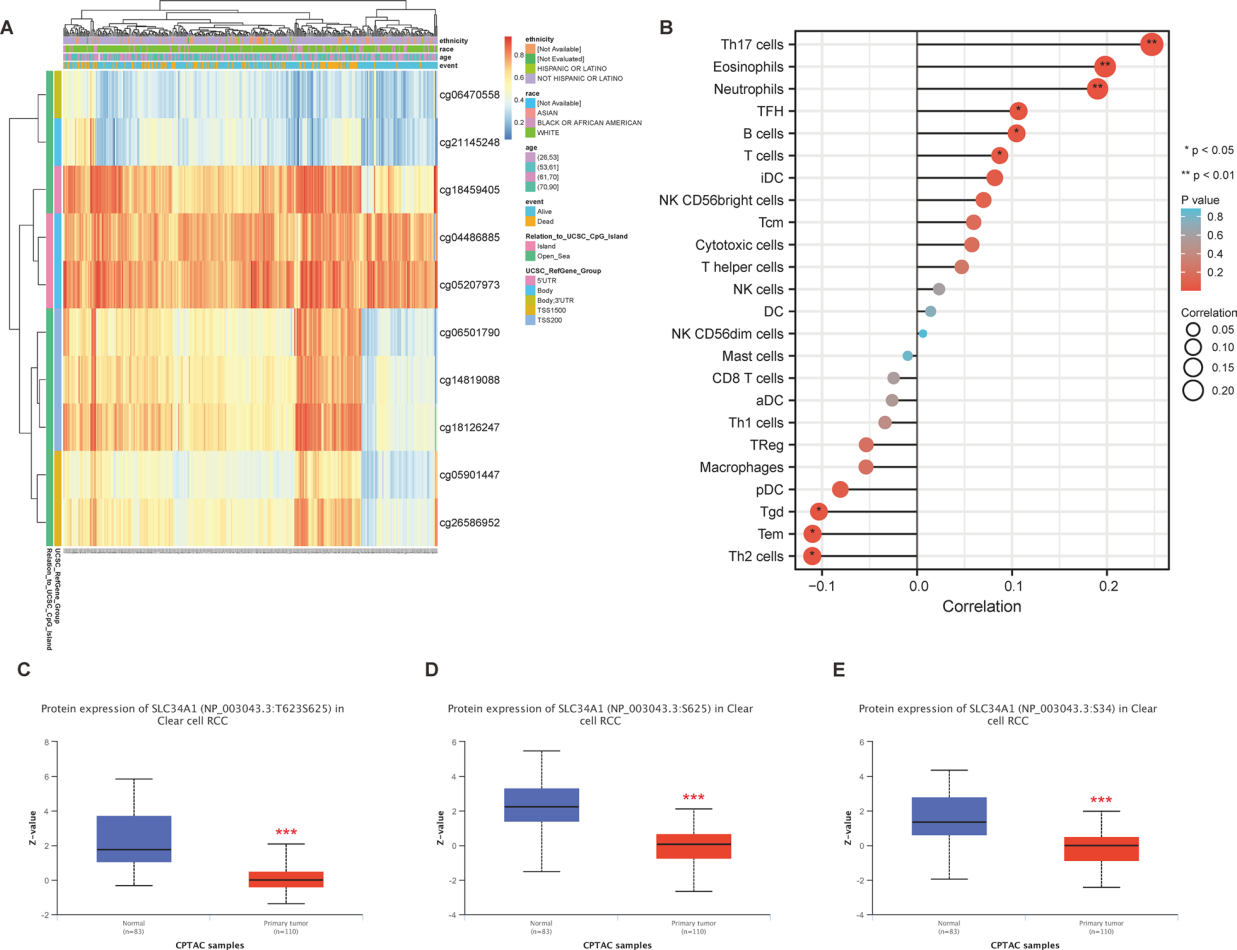
Methylation level and phosphoprotein expression of SLC34A1 in ccRCC and its correlation with immune cell infiltration. **A** Visualization of SLC34A1 methylation levels. **B** Correlation analysis showing that SLC34A1 is positively associated with the infiltration of B cells, eosinophils, neutrophils, T cells, TFH, and Th17 cells, and negatively associated with the infiltration of Tem, Tgd, and Th2 cells. **P < 0.01and *P < 0.05 were both statistically significant. **C**–**E** CPTAC analysis of SLC34A1 phosphoprotein expression at T623S625, S625, and S34 sites in primary ccRCC tumors and normal renal tissues. ***P < 0.001 was statistically significant

We evaluated the methylation level of SLC34A1 and the impact of each CpG on ccRCC prognosis using the MethSurv online tool. Our analysis showed that SLC34A1 has 10 methylation sites, with the highest methylation levels observed at cg04486885 and cg05207973 (Fig. 10A). Of the 8 methylation sites associated with prognosis, namely cg05901447, cg06470558, cg06501790, cg14819088, cg18126247, cg18459405, cg21145248, and cg26586952 (Table 6), patients with high SLC34A1 methylation levels at these sites exhibited worse overall survival compared to those with low methylation levels. We also found that SLC34A1 was positively correlated with the infiltration levels of B cells, eosinophils, neutrophils, T cells, TFH, and Th17 cells, and negatively correlated with the infiltration levels of Tem, Tgd, and Th2 cells (*P* < 0.05) (Fig. 10B). Using the CPTAC database, we analyzed the expression of SLC34A1 phosphoprotein in ccRCC. Our results revealed that the expression of SLC34A1 phosphoprotein at the T623S625, S625, and S34 phosphorylation sites was significantly lower in primary ccRCC tumors than in normal renal tissues (*P* < 0.001) (Fig. 10C–E).

**Table 6:** Effects of hypermethylation level of SLC34A1 on the prognosis of ccRCC

CpG	HR	*P* value
cg04486885	0.76	0.252742
cg05207973	1.269	0.224987
cg05901447	3.478	0.00035
cg06470558	1.851	0.023503
cg06501790	6.611	7.18E−06
eg14819088	3.098	0.000388
eg18126247	7.371	1.31E−05
eg18459405	3.126	0.000351
cg21145248	3.443	0.00011
cg26586952	3.567	0.000265

### Expression verification of SLC34A1 in clinical samples

**Fig. 11:**
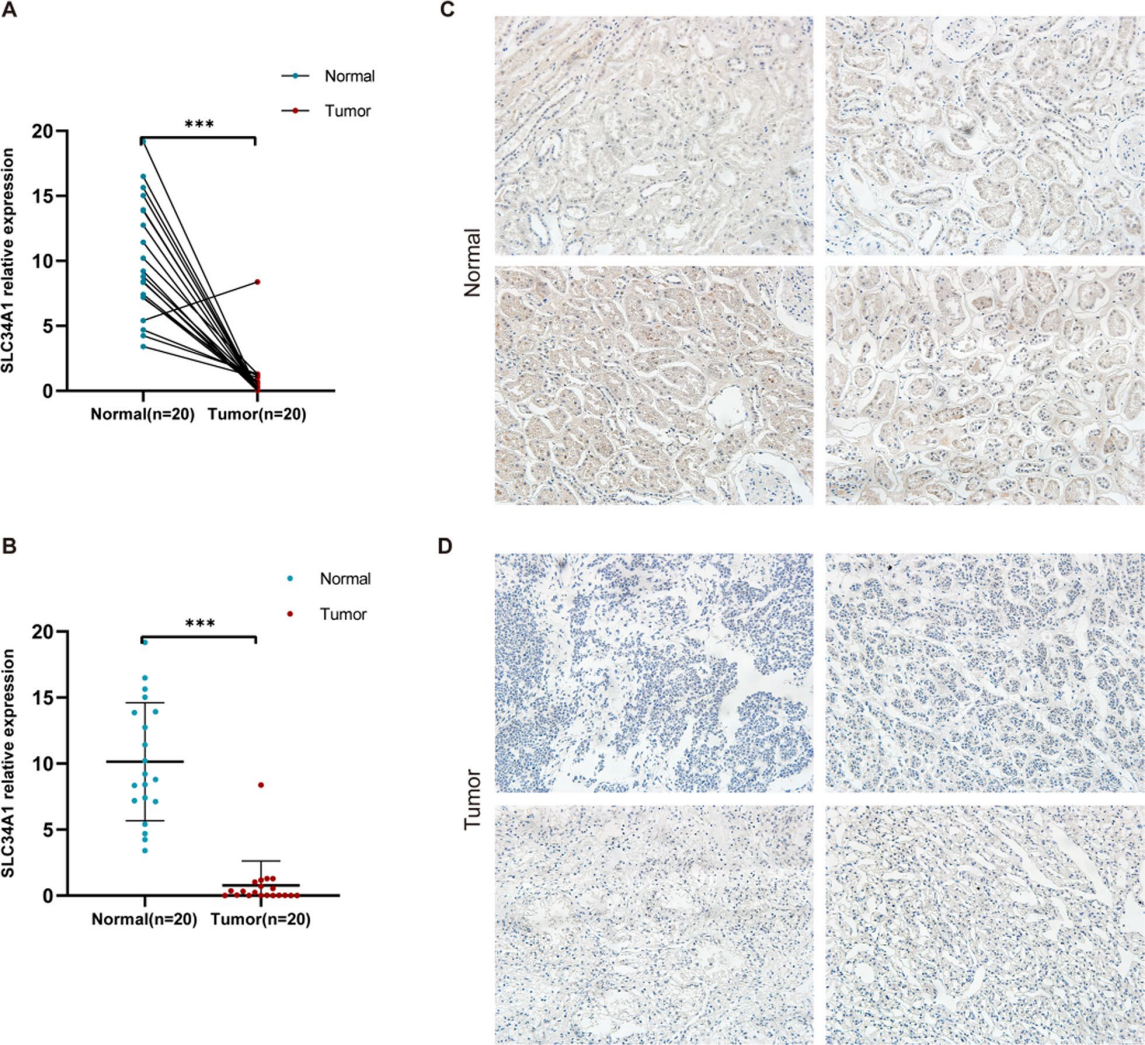
Expression of SLC34A1 in clinical samples. **A**–**B** qRT-PCR results showed a significant decrease in SLC34A1 expression in clinical samples. **C**–**D** IHC assay revealed lower SLC34A1 protein expression in ccRCC tissues compared to adjacent normal tissues (× 200 magnification)

To verify the expression of SLC34A1, we used qRT-PCR and IHC assay to detect the expression level of SLC34A1 in ccRCC tissues. The results confirmed that SLC34A1 was significantly decreased in ccRCC tissues (Fig. 11).

## Discussion

Renal cell carcinoma is a highly prevalent form of cancer in humans, with over 338,000 new cases reported globally in 2012, accounting for 24% of all malignancies. It is estimated that 143,000 people died from kidney cancer that year, making it the 16th leading cause of cancer-related deaths worldwide [23]. Due to its complex molecular mechanism and insensitivity to traditional cancer therapies, it is crucial to gain a deeper understanding of the molecular mechanisms of ccRCC, improve risk assessment, guide clinical decision-making, and enhance the diagnosis, treatment, and prognosis of this disease.

Recently, bioinformatic analyses based on gene expression microarrays have provided valuable insights into the pathogenesis of ccRCC and identified potential diagnostic and therapeutic targets by collecting and analyzing relevant data [24]. From six datasets in the GEO database, a total of 58 overlapping DEGs (8 up-regulated genes and 50 down-regulated genes) were identified. The five genes with the highest scores were selected as hub genes in the PPI network: SLC34A1, KCNJ1, SLC12A3, CASR, and ALDOB. Further analysis using the GEPIA online tool revealed that ccRCC patients with low levels of SLC34A1, CASR, and ALDOB had poorer overall survival. Additionally, low SLC34A1, CASR, and ALDOB expressions were associated with poorer DFS.

ALDOB encodes for aldolase B, a protein primarily expressed in the liver and kidney that plays a role in glycolysis and fructose decomposition [25]. Low ALDOB expression has been linked to various diseases, including hepatocellular carcinoma, colon adenocarcinoma, and hereditary fructose intolerance (HFI). For example, Liu et al. [26] found that aldob downregulation can activate the upregulation of IR signal and adipogenesis in human HCC tumor tissue to promote the occurrence of HCC. Research has also shown that low ALDOB expression is associated with poor prognosis in ccRCC [27], consistent with the findings of the current study.

Calcium-sensitive receptor (CASR), a member of the G-protein-coupled receptor (GPCR) family, is mainly expressed in the parathyroid gland and renal tubules. Abnormalities in CASR can cause various diseases, including familial hypocalcemia, hypercalcemia, autosomal dominant hypocalcemia, and V-type Bart syndrome [28, 29]. CASR's role in promoting or inhibiting tumor development varies depending on the type of cancer. Li et al. [30] Found that CASR may pass GSK3β/Cyclin, inhibiting the development of human lung adenocarcinoma. In contrast, in gastric cancer, Xie et al. [31] believe that CASR is related to the tumor progression of gastric cancer and the poor survival rate of these patients. Unfortunately, although some studies have pointed out that the expression level of CASR in primary ccRCC is very low when metastatic RCC expresses CASR, extracellular calcium will promote the cell migration and proliferation of bone metastatic RCC cells through CASR and its downstream signaling pathway [32]. Therefore, we cannot use CASR as a marker of a positive prognosis.

The influence of KCNJ1 and SLC12A3 among the five hub genes on DFS in ccRCC patients was not statistically significant, and CASR would lead to the migration and proliferation of metastatic RCC cells. The literature review found that the detailed mechanism of ALDOB inhibiting the progression of renal cell carcinoma has been confirmed by other scientists [33]. Finally, the author decided to select SLC34A1 for further analysis and research. This involved studying the relationship between SLC34A1 and its related gene mutations, phosphoprotein expression, DNA methylation, and immune cell infiltration to explore the value of SLC34A1 in the occurrence and development of renal cell carcinoma.

SLC is a family of membrane-binding proteins, consisting of more than 300 proteins that facilitate the transport of various substrates across biofilms [34]. SLC34A1 is responsible for coordinating the transport of sodium phosphate by regulating phosphate reabsorption in proximal tubules [35]. Autosomal recessive mutations in SLC34A1 can lead to idiopathic infantile hypercalcemia [36]. In our study, we observed that the mRNA and protein expression of SLC34A1 was significantly downregulated in ccRCC samples compared to normal samples. SLC34A1 expression displayed strong discriminatory power in distinguishing ccRCC tumors from normal tissues. Additionally, SLC34A1 exhibited significant correlation with clinicopathological features at the transcriptome level, such as age, gender, T stage, pathological stage, and M stage. Furthermore, SLC34A1 was identified as an independent predictive factor in both univariate and multivariate analyses for ccRCC patients.

Analysis of SLC34A1-related genes revealed that most of these genes are involved in biological processes such as immune response-regulating signaling pathways, organic cation transport, and regulation of innate immune response. The five genes with the strongest correlation to SLC34A1 are associated with better prognosis in patients with ccRCC. SLC5A10 is a sodium-dependent transporter that may be involved in decreasing serum 1,5-AG levels in diabetic patients [37]. However, its potential mechanism is still not well understood due to limited research. AGPAT3 is an enzyme related to lipid metabolism that promotes DHA accumulation in the brain, providing DHA-PLs [38]. It is also highly expressed in gastric cancer patients with good prognosis [39]. ATP6V1B2 encodes the subunit of V-ATPase, which is responsible for lysosomal acidification [40]. Abnormality in ATP6V1B2 can lead to Zimmermann–Laband syndrome and dominant deafness onychodystrophy syndrome [41, 42]. SLC35D2 is a member of the SLC35 nucleotide sugar transporter family and plays an essential role in the synthesis of glycosaminoglycans (GAGs) [43]. CIRH1A is a human ribosomal protein, and a homologous missense mutation at the C-terminal of CIRH1A can lead to cirrhosis (NAIC) in North American Indian children [44]. It is abundant in colon cancer and enhances the development of RKO colorectal cancer cells [45]. Based on previous experimental data and our survival analysis, these genes may be potential candidate genes for inhibiting cancer development.

In 643 ccRCC patients, the genetic change frequency of the SLC34A1 gene was 13%, and most SLC34A1 changes were represented by amplification, indicating a close association between SLC34A1 mutation and ccRCC. The top five genes with the highest frequency in the SLC34A1 change group were B4GALT7, DOK3, FAM193B, HK3, and PDLIM7. Furthermore, changes in SLC34A1 were associated with changes in VHL and BAP1, which are factors that inhibit the progression of ccRCC. DNF methylation occurs in almost all cancers as a common modification mechanism. In our study, we examined the association between SLC34A1 DNA methylation and the prognosis of ccRCC patients, and found that hypermethylation of eight CpG sites was associated with poor overall survival.

The tumor microenvironment has a significant impact on cancer occurrence, development, and metastasis, with tumor-associated immune cells being a crucial part of it. Currently, clinical therapy utilizes cytokine and immune checkpoint inhibitors [46, 47]. This study found a correlation between SLC34A1 and various immune cells, including B cells, eosinophils, neutrophils, T cells, TFH, and Th17 cells, with positive correlation, and Tem, Tgd, and Th2 cells, with negative correlation. Eosinophils have been linked to a favorable prognosis of ccRCC and are an independent predictor of nivolumab treatment in metastatic patients [48]. Our findings also suggest that SLC34A1 may play a crucial role in immune regulation, as it was positively correlated with Th17 cells and negatively correlated with Th2 cells. Conversely, higher levels of T17 cells were correlated with improved survival rates in ccRCC patients, while Th2 cells were associated with negative results [49]. These results indicate that SLC34A1 could reflect the state of the ccRCC immune microenvironment and could be a potential diagnostic and predictive biomarker, as well as a treatment option for ccRCC. In addition, clinical samples were used to verify that the mRNA and protein expression levels of SLC34A1 in ccRCC tissues were significantly lower than those in normal kidney tissues.

We analyzed the potential hub genes and signaling pathways involved in the development of ccRCC using DEGs of ccRCC and normal tissues. Furthermore, we verified the value of SLC34A1 in the diagnosis and prognosis of ccRCC. SLC34A1 DNA methylation was found to be related to ccRCC prognosis. In conclusion, our study suggests that SLC34A1 has the potential to be a diagnostic and prognostic marker for ccRCC and could be a target for clinical diagnosis, prognosis, and treatment. However, our study has limitations, including the need to expand the sample size to increase the credibility of our results and to conduct more basic research to validate our findings and promote clinical applications.

## Conclusions

The results of this study show that the expression level of SLC34A1 is significantly lower in ccRCC. This suggests that SLC34A1 may serve as a valuable diagnostic and prognostic marker for ccRCC, and that SLC34A1 DNA methylation could be linked to the prognosis of ccRCC patients. Further research is needed to explore the potential of SLC34A1 as a target for the clinical diagnosis, prognosis, and treatment of ccRCC. In particular, more basic research is required to validate these findings and promote their clinical application.

## Supplementary Information

**Additional file 1**: **Fig. S1**. Work flowchart

## Data Availability

Six data sets including GSE66272 (https://www.ncbi.nlm.nih.gov/geo/query/acc.cgi?acc=GSE66272), GSE53757 (https://www.ncbi.nlm.nih.gov/geo/query/acc.cgi?acc=GSE53757), GSE168845 (https://www.ncbi.nlm.nih.gov/geo/query/acc.cgi?acc=GSE168845), GSE40435 (https://www.ncbi.nlm.nih.gov/geo/query/acc.cgi?acc=GSE40435), GSE96574 (https://www.ncbi.nlm.nih.gov/geo/query/acc.cgi?acc=GSE96574), and GSE68417 (https://www.ncbi.nlm.nih.gov/geo/query/acc.cgi?acc=GSE68417) were obtained from GEO database, and RNA sequencing and clinical data were obtained from TCGA database. Further information can be obtained from Jiechuan Qiu if necessary.
