# Supplementary material for: Low expression of SLC34A1 is associated with poor prognosis in clear cell renal cell carcinoma
Source: BMC Urol. 2023 Mar 28;23:45. doi: 10.1186/s12894-023-01212-x (PMC10044763; doi:10.1186/s12894-023-01212-x)
Supplement: Supplementary file 1 — Additional file 1: Fig. S1. Work flowchart [file 12894_2023_1212_MOESM1_ESM.docx]

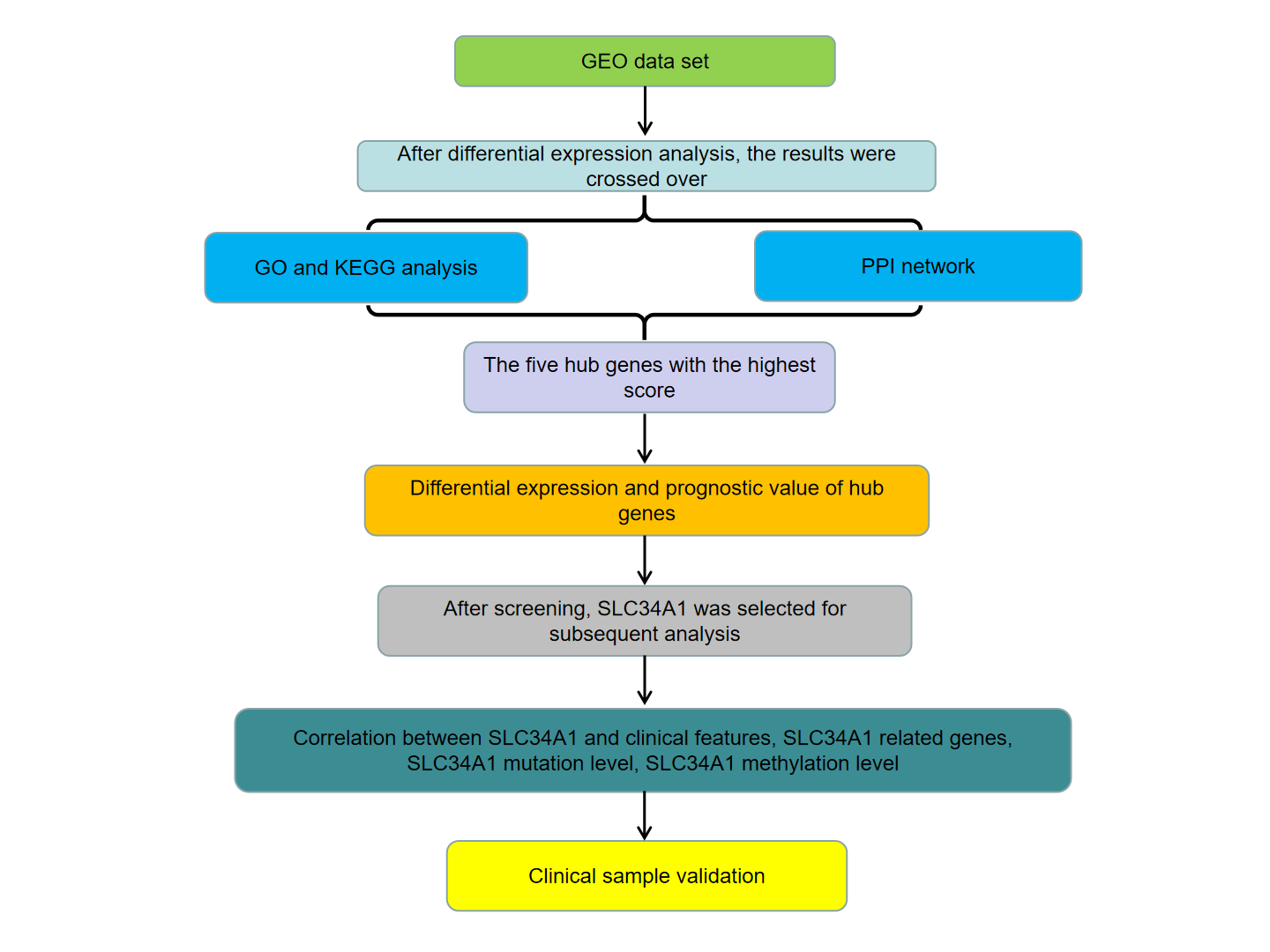


**Supplementary Figure 1** Work flowchart

The detailed research process of this study is presented in the flowchart.
